# End‐Of‐Life Outcomes and Healthcare Utilization for Patients With Hepatocellular Carcinoma Who Received Immune Checkpoint Inhibition

**DOI:** 10.1002/cam4.71293

**Published:** 2025-10-10

**Authors:** Margaret C. Wheless, Anna‐Carson R. Uhelski, Sarah K. Cimino, Henry J. Domenico, Sara F. Martin, Mohana B. Karlekar, Thatcher R. Heumann, Kristen K. Ciombor, Laura W. Goff, Rajiv Agarwal

**Affiliations:** ^1^ Vanderbilt University Medical Center Nashville Tennessee USA; ^2^ Vanderbilt Ingram Cancer Center Nashville Tennessee USA

**Keywords:** end‐of‐life, goals of care, hepatocellular carcinoma, immune checkpoint inhibitors

## Abstract

**Introduction:**

Immune checkpoint inhibitors (ICI) have revolutionized treatment for advanced hepatocellular carcinoma (HCC). However, end‐of‐life (EOL) outcomes and healthcare utilization patterns prior to death for patients who receive ICI compared to non‐ICI as their last therapy are unknown.

**Methods:**

Patients with advanced HCC evaluated from January 1, 2020, and who died by March 29, 2024, were included for analysis. Primary EOL outcomes include: advance directives, goals of care conversations, location of death, palliative care referral, hospice referral, and hospice duration. Secondary healthcare utilization outcomes include systemic therapy receipt, emergency department visits, hospitalizations, and intensive care unit admissions within 14, 30, and 90 days of death. Outcomes were stratified by ICI or non‐ICI as the last therapy received, and *p* values were derived using Pearson's chi‐square test for equality of proportions.

**Results:**

We identified 71 patients for our retrospective cohort: mean age 64.1 years; 70.8% male; Child Pugh (CP) status at last treatment: 51.1% CPA, 40.0% CPB, 8.9% CPC. No differences in EOL outcomes were detected between groups; median days enrolled in hospice did not differ (24.5 days [non‐ICI] vs. 10 days [ICI]; *p* = 0.39). Yet, a higher proportion of patients who received ICI as the last therapy had increased healthcare utilization across secondary outcomes.

**Conclusions:**

Patients with advanced HCC receiving ICI as their last treatment before death, compared to those receiving non‐ICI, had similar EOL outcomes but higher healthcare utilization. Further investigation into risk stratification to predict high healthcare utilizers could guide decision‐making around the ongoing use of ICI near the EOL.

## Abstract 2

Patients with hepatocellular carcinoma received more immune checkpoint inhibitors (ICI) closer to death than patients treated with non‐ICI therapy. Patients who received ICI as their last therapy had higher healthcare utilization at the end of life.

## 1 Introduction

With the development and incorporation of immune checkpoint inhibitors (ICI) into the treatment algorithm of hepatocellular carcinoma (HCC), the treatment paradigm has shifted drastically in the last several years, particularly for patients with Child Pugh A (CPA) cirrhosis. Since the U.S. Food and Drug Administration approval of both atezolizumab/bevacizumab (atezo/bev) in 2020, based on the IMbrave150 trial [1] and durvalumab/tremelimumab (durva/treme) in 2022 after the HIMALAYA trial [2], ICIs have become the standard of care for qualifying patients with advanced HCC in the first‐line setting [3]. In the second‐line, the phase I/II Checkmate‐040 trial showed efficacy of ipilimumab/nivolumab (ipi/nivo) for patients previously treated with tyrosine kinase inhibitors (TKIs) [4]. Checkmate‐040 notably included a cohort of Child Pugh B7 and B8 patients who received nivo monotherapy with an acceptable safety profile and clinical efficacy [5]. Recently, results from the phase III Checkmate‐9DW trial comparing ipi/nivo to TKI as first‐line treatment for patients with advanced HCC confirmed improved outcomes with front‐line ICI compared to TKIs in CPA patients [6]. ICIs are generally viewed as better tolerated than traditional chemotherapy or TKIs and, in some cases, can lead to durable responses. Because of their favorable side effect profile, their use may be increasing in patients with HCC with progressive disease and who are nearing the end of life with the aim to achieve a therapeutic response while maintaining quality of life (QOL) [7].

In several other solid tumor types, the utilization of ICI close to death has been previously examined. For example, following the approval of ICI, the use of systemic therapy within 30 days of death for patients with metastatic melanoma increased [8]. This increase was similarly seen in urothelial cancer after ICI approval [9, 10]. In comparing patients with high‐risk melanoma and poor prognosis to those with good prognosis, there were similar rates of hospice referrals between the two cohorts. Interestingly, ICI use was more common in patients with high‐risk melanoma, suggesting that oncologists often give ICI while realizing patients may be nearing the end of life [10]. Yet, in patients with advanced, high‐risk melanoma, up to 34% lived more than 1 year with ICI alone, suggesting that certain patients can still benefit from treatment [10]. Unfortunately, it remains unclear how to predict which patients may benefit from ICI in various solid tumors, particularly for those with high‐risk disease who may be near the end of life.

Child‐Pugh status has been widely adopted as a reliable way to predict prognosis in patients with cirrhosis with 5 year overall survival (OS) rates ranging from 61% in CPA to 25% in Child‐Pugh C (CPC) [11]. Because most ICI studies in HCC include only patients with CPA status, the real‐world utilization, outcomes, and safety of ICI in Child‐Pugh B (CPB) patients are less known. A retrospective meta‐analysis of 22 studies comparing adverse events (AEs) in HCC patients with CPA compared to CPB status treated with ICI showed similar rates of immune‐related adverse events (irAEs) [12]. The rates of irAEs were also similar in the CPB cohort of CheckMate‐040, which included CPB patients treated with nivo [5]. In a retrospective analysis including 65 patients with CPA (*n* = 32), CPB (*n* = 28), and Child‐Pugh C (CPC) (*n* = 5) who received ICI in the 1st, 2nd, and 3rd line of treatment, AEs were comparable between CPA and CPB patients [13]. It is notable that OS in this study is significantly shorter in the CPB patients compared to CPA (8.6 months vs. 16.7 months; *p* = 0.065), likely a reflection of clinical decline secondary to underlying liver dysfunction, which may confound the true efficacy of ICIs [13]. Even within CPB patients, prognosis significantly varies between CPB7 and CPB8/9 patients. A retrospective study evaluating patients treated with atezo/bev found that the median OS (mOS) in CPB7 patients was 9.1 months compared to just 5.5 months and 4.0 months in CPB8 and CPB9 patients, respectively. Similarly, the median progression‐free survival (mPFS) of 6.4 months in CPB7 patients was more comparable to the CPA cohort (8.9 months) than CPB8/9 cohort (2.7 months) [14]. Not only do these results highlight the heterogeneity of CPB patients, but also emphasize that outcomes in CPB8/9 patients are likely driven by liver dysfunction, and these patients are less likely to derive benefit from systemic therapy [14].

End‐of‐life (EOL) outcomes for patients with HCC across various degrees of hepatic function who receive ICI compared to other approved therapies, such as TKIs prior to death are unknown. The few prior studies examining EOL outcomes in patients with HCC were done prior to the approval of ICI, and to our knowledge, this study is unique in reporting both EOL and healthcare utilization outcomes in the era of immunotherapy for treatment of HCC. One study prior to ICI use, done between 2006 and 2012 reported on 82 patients with HCC; 71% were hospitalized, 48% had emergency room visits, and 12% had intensive care unit admissions within the last month of life [15]. Another study prior to ICI approval evaluating the impact of palliative care interventions on cost and EOL care found that procedures were reduced during terminal hospitalizations with palliative care involvement, decreasing cost [16]. Neither of these studies has examined the effects of both EOL outcomes and healthcare utilization in patients who received ICI compared to the historical standard of care therapy. As TKIs have been the standard of care and control treatment in immunotherapy trials, we stratified patients by receipt of ICI or non‐ICI (e.g., TKI or chemotherapy to include real‐world data). Herein, we examine relationships between EOL outcomes, healthcare utilization, and receipt of ICI for patients with advanced HCC.

## 2 Methods

### 2.1 Data Collection

**FIGURE 1:**
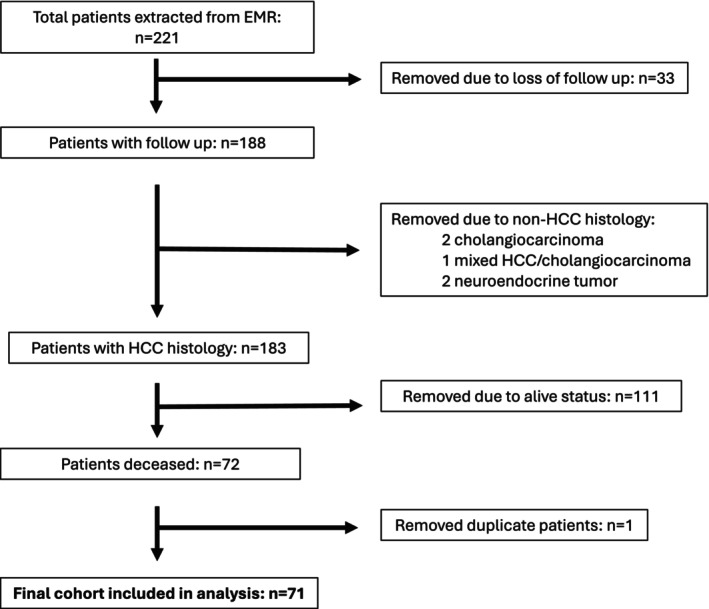
Patient selection. Patient selection process is shown. Initially, data from 221 patients were extracted from the EMR based on diagnostic codes of liver cancer and hepatocellular carcinoma. These diagnoses were manually reviewed, and patients with non‐HCC histology and duplicates were excluded. All patients who were deceased by the data cutoff date of 3/29/24 were included in the final analysis. Abbreviations: EMR, electronic medical record; HCC, hepatocellular carcinoma.

All patients referred to our tertiary cancer center, Vanderbilt Ingram Cancer Center, who were evaluated by a medical oncologist between January 1, 2020, and January 1, 2024, with a diagnosis of advanced HCC, were screened for inclusion in this study. The database of patients was developed using the electronic medical record (EMR) by including diagnostic codes of liver cancer and hepatocellular carcinoma, and these diagnoses were subsequently verified with manual chart review to confirm that patients had a diagnosis of HCC (Figure 1). All patients who were deceased by a data cutoff date of 3/29/2024 were included in the final analysis after approval by the institutional review board.

Patient information was collected by manual extraction of data from the EMR which included: gender; age at diagnosis; Child‐Pugh status at diagnosis and at time of last systemic treatment (either extracted from clinic notes or manual calculation based on labs and clinical factors noted at the time of diagnosis and time of last systemic treatment); age at the time of last systemic treatment; ICI as last treatment; prior locoregional therapy (including type and number of treatments); prior HCC surgery or liver transplant; etiology of cirrhosis; MELD‐Na score at the time of last systemic treatment (either extracted from notes or manual calculation based on labs and clinical factors noted at the time of last treatment); lines of prior treatment and the reason for their discontinuation; the presence of grade 3 or 4 irAEs (including type of irAE); ECOG performance status as documented in notes upon referral and at initiation of last treatment. Last systemic treatment is defined as the last line of systemic therapy the patient received for at least one dose at any time point prior to death.

Primary EOL outcomes were collected by manual extraction from the EMR and included advance directives (AD) documented prior to death, documentation of GOC conversations, death in a hospital or facility, palliative care referral prior to death, hospice referral prior to death, hospice duration in days, and DNR/DNI status prior to death. Hospice duration was determined by the time elapsed from hospice enrollment to the date of death. Our institution encourages the use of a standardized advanced care planning template within the EMR; however, consistent implementation of this template in clinical practice is not guaranteed, and accordingly, this can lead to variability. Moreover, the content and documentation of such discussions may vary among oncology providers. Therefore, our independent review was less restrictive and included a review of both templated and nontemplated language. This included review of completion of advance directives, and review of discussions documentation by oncologists under the heading “Goals of Care,” which included discussions relating to personal values and goals surrounding EOL care, advance care planning, illness understanding, and life‐sustaining treatment preferences. Patients whose EOL care was provided exclusively by our institution but who did not have AD documentation, GOC conversations, hospice, or palliative care referral within our EMR were classified as “known” without any of those outcomes. Patients who received EOL care at our facility and an outside facility who did not have AD documentation, GOC conversations, hospice, or palliative care referral within our EMR were classified as “unknown,” since these outcomes could have taken place at an outside institution.

Secondary EOL and healthcare utilization outcomes were manually extracted from the EMR and include: receipt of systemic therapy, hospitalization, intensive‐care unit (ICU) admission, and emergency department (ED) visits within 14 days, 30 days, and 90 days of death. Patients whose EOL care was provided exclusively by our institution and did not have documentation of hospitalization, ICU admission, or emergency department visit were classified as “known” without any of those outcomes. For patients who received EOL care at our facility and an outside facility who did not have any documentation of hospitalization, ICU admission, or emergency department visit within our EMR, were classified as “unknown,” since these outcomes could have taken place at an outside institution.

### 2.2 Statistical Analysis

Differences in EOL outcomes and healthcare utilization outcomes were stratified based on whether ICI or non‐ICI treatment was administered as the last therapy prior to death. Pearson's chi‐square test was used to assess differences in proportions between groups. Additionally, hospice duration between the two groups was compared using a Wilcoxon rank sum test. Healthcare utilization outcomes, stratified by last therapy (ICI vs. non‐ICI), were summarized using counts and proportions. A comparison of the proportion of patients receiving ICI as last therapy among all utilization outcome groups at 90, 30, and 14 days before death was performed using the nonparametric Pearson's chi‐square test; no testing for normality was performed.

Details regarding the type of ICI administered and rates of irAEs were summarized using counts and proportions. Additionally, the receipt of ICI or non‐ICI therapy within 90, 30, and 14 days of death was further stratified by Child‐Pugh classification at the time of last treatment.

## 3 Results

### 3.1 Patient Characteristics

Using diagnostic codes, 221 patients' data were extracted from the EMR, of which 33 were lost to follow‐up. Of the 188 remaining patients, six patients were removed (five did not have a diagnosis of HCC, and one patient was removed as a duplicate upon manual chart verification). As of the data cutoff date of March 29, 2024, 111 patients were still living, and 71 deceased patients were included in the final analysis (Figure 1).

Table 1 summarizes patient characteristics in the cohort. The mean age was 64.1 years, and 70.8% were male. The most common etiology for cirrhosis was HCV (45.5%), followed by alcoholic cirrhosis (26.3%) and nonalcoholic liver disease (21.2%). Of the 71 patients, the number of lines of therapy received was known in 81.7% (*n* = 58), of whom seven patients received no treatment, 32 received one line of therapy, 13 received two lines of therapy, five received three lines of therapy, and one received four or more lines of therapy. At diagnosis of HCC, 54 (79.4%) patients were CPA, 14 (20.6%) were CPB, and no patients were CPC. This differed from CP status at the time of last treatment, at which 23 (51.1%) were CPA, 18 (40.0%) were CPB, and 4 (8.9%) were CPC. The Eastern Cooperative Oncology Group Performance Status (ECOG PS) was collected at the time of referral and at the time of initiation of the last treatment. Median ECOG PS was 1 (range 0–3) at both time points.

**TABLE 1:** Patient characteristics.

Patient characteristics, *n* = 71		*n* (%)
Gender	Male	51 (70.8)
	Female	20 (28.2)
Age at diagnosis	Mean (range) in years	64.1 (35–83)
Age at last systemic therapy	Mean (range) in years	64.2 (35–84)
Lines of systemic therapy	*n* (%)	58 (81.7)
	Number of lines; mean (range)	1.59 (1–7)
	Number of lines, median	1
Prior locoregional therapy	*n* (%)	31 (43.7)
	Number of treatments; mean (range)	1.72 (1–10)
	Arterially directed therapy	26 (83.9)
	External beam radiation	3 (9.7)
	Ablation	5 (16.1)
Prior surgery for HCC	Yes	11 (15.7)
		1 unknown
Prior liver transplant	Yes	3 (4.3)
		2 unknown
CP status at diagnosis	*n* (% known)	68 (95.8)
	CPA5	38 (55.9)
	CPA6	16 (23.5)
	CPB7	4 (5.9)
	CPB8	6 (8.8)
	CPB9	4 (5.9)
	CPC10	0 (0)
CP status at initiation of last treatment	*n* (% known)	45 (63.4)
	CPA5	13 (28.9)
	CPA6	10 (22.2)
	CPB7	8 (17.8)
	CPB8	4 (8.9)
	CPB9	6 (13.3)
	CPC10	4 (8.9)
ECOG PS at referral	*n* (% known)	64 (90.1)
	0	25 (39.1)
	1	28 (43.8)
	2	8 (12.5)
	3	3 (4.7)
	Median (range)	1 (0–3)
ECOG PS at initiation of last treatment	*n* (% known)	46 (64.8)
	0	6 (13.0)
	1	27 (58.7)
	2	11 (23.9)
	3	2 (4.3)
	Median (range)	1 (0–3)
MELD‐Na, initiation of last treatment	Mean (range)	14.2 (6–34)
Etiology of cirrhosis	*n* (% known)	66 (93.0)
	EtOH	15 (22.7)
	NASH	14 (21.2)
	HCV	30 (45.5)
	HBV	3 (4.5)
	Hemochromatosis	0 (0)
	Other	6 (9.1)
	No cirrhosis	9 (13.6)

*Note:* Table 1: Patient characteristics are shown. Any *n* is assumed to be the total number of patients, *n* = 71, unless otherwise specified for missing data. Arterially directed therapy includes transarterial chemoembolization (TACE) and Y90 therapy.

Abbreviations: CP, Child‐Pugh; ECOG PS, Eastern Cooperative Oncology Group Performance Status; EtOH, alcohol; HBV, hepatitis B virus; HCC, hepatocellular carcinoma; HCV, hepatitis C virus; NASH, nonalcoholic steatohepatitis.

### 3.2 Primary Outcomes

Primary EOL outcomes are summarized in Table 2. Among the 71 patients included, 31.0% had AD documented prior to death. There was no difference in AD documentation rates (44.8% vs. 39.1%; *p* = 0.68) or GOC conversation documentation (79.2% vs. 85.2%; *p* = 0.57) in patients who received ICI versus non‐ICI therapy as their last treatment. Eighteen patients' locations of death were known, of which 35.7% of patients who received ICI last compared to 38.1% of patients who received non‐ICI therapy last died in a hospital or facility (*p* = 0.86). The proportions of patients who had palliative care (50.3% ICI vs. 69.2% non‐ICI; *p* = 0.48) or hospice referrals (59.2% ICI vs. 87.0% non‐ICI; *p* = 0.31) were not statistically different based on the type of last therapy. The median number of days enrolled in hospice was 24.5 days (mean 71.3 days; interquartile range [IQR] 3.5–60.0) for patients who did not receive ICI as last treatment and 10 days (mean 41.8 days; IQR 3.5–45.5) for patients who received ICI (*p* = 0.39). Of the 46 patients whose code status was known, 89.3% of the patients who received ICI last were DNR/DNI, and 91.3% of patients receiving non‐ICI therapy were DNR/DNI (*p* = 0.81).

**TABLE 2:** End‐of‐life outcomes by receipt of immune checkpoint inhibition.

Outcome	Total *n* (%) *n* = 71	Last treatment: ICI *n* (% of known)	Last treatment: non‐ICI *n* (% of known)	*p*
AD documented				
Yes	22/71 (31.0)	13/29 (44.8)	9/23 (39.1)	0.68
No	35/71 (49.3)	16/29 (55.2)	14/23 (60.9)	
Unknown	14/71 (19.7)			
GOC discussion documented				0.57
Yes	42 (59.2)	19/24 (79.2)	23/27 (85.2)	
No	15 (21.1)	5/24 (20.8)	4/27 (14.8)	
Unknown	14 (19.7)			
Death in hospital or facility				
Yes	18/71 (25.4)	10/28 (35.7)	8/21 (38.1)	0.86
No	32/71 (45.1)	18/28 (64.3)	13/21 (61.9)	
Unknown	21/71 (29.6)			
Palliative care referral				
Yes	36/71 (50.7)	18/30 (60.0)	18/26 (69.2)	0.47
No	22/71 (31.0)	12/30 (40.0)	8/26 (30.8)	
Unknown	13/71 (18.3)			
Hospice referral				
Yes	42/71 (59.2)	22/29 (75.9)	20/23 (87.0)	0.31
No	11/71 (15.5)	7/29 (24.1)	3/23 (13.0)	
Unknown	18/71 (25.4)			
DNR/DNI prior to death				
Yes	46/71 (64.8)	25/28 (89.3)	21/23 (91.3)	0.81
No	7/71 (9.9)	3/28 (10.7)	2/23 (8.7)	
Unknown	18/71 (25.4)			

*Note:* Table 2 End‐of‐life outcomes stratified by receipt of immune checkpoint inhibitors prior to death. Percentages of patients who received ICI as their last treatment are represented as percentages of known outcomes. Any treatment other than ICI (TKI or chemotherapy) was counted as part of non‐ICI treatment. *p* values are derived using Pearson's chi‐square test for equality of proportions.

Abbreviations: AD, advance directive; GOC, goals of care; ICI, immune checkpoint inhibitor.

### 3.3 Secondary Outcomes

**FIGURE 2:**
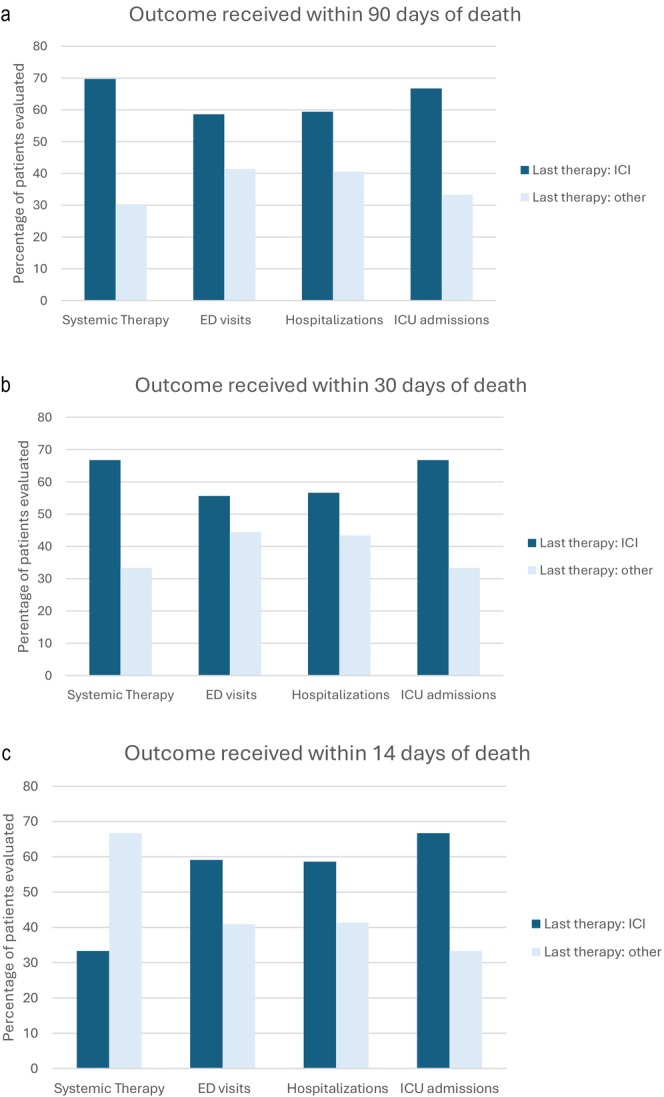
Healthcare Utilization Outcomes Relative to ICI versus non‐ICI Therapy Prior to Death. Figure 2a–c demonstrate outcomes received within 90, 30, or 14 days of death stratified by ICI versus non‐ICI as the last therapy, respectively. Healthcare utilization outcomes included ED visits, hospitalizations, and ICU admissions, which were all increased across 90‐, 30‐, and 14‐day time‐periods prior to death in patients who received ICI as the last therapy. Patients did not have to receive ICI during the 90, 30, or 14 days prior to death, but did have to receive ICI as their last treatment to be included in the ICI cohort. P‐value for difference in proportion of patients with last therapy ICI between utilization outcome group at 90 days = 0.780, 30 days = 0.88, and 14 days = 0.64.

Secondary healthcare utilization outcomes are summarized in Figure 2 and Table S1. There was a greater proportion of patients who received ICI versus non‐ICI therapy within 90 and 30 days of death (69.7% and 66.7%, respectively). Our results also show that a greater proportion of patients received ICI as their last systemic therapy, regardless of the time from last ICI to healthcare outcome for the remaining healthcare utilization outcomes, and within the above timeframes relative to death. Moreover, there was no difference in the proportion of patients with last therapy ICI among these healthcare utilization outcomes at 90, 30, and 14 days (*p* = 0.780, 0.88, and 0.64, respectively). For the 29 patients who presented to an ED within 90 days of death, 58.6% had received ICI as their last‐line therapy compared to other therapies, and this majority held true for ED visits within 30 days (55.6%) and 14 days (59.1%) of death. Similarly, most patients who were hospitalized within 90, 30, and 14 days of death (59.4%, 56.6%, and 58.6%, respectively), and 66.7% of patients admitted to the ICU within these timeframes received ICI as their last therapy.

**FIGURE 3:**
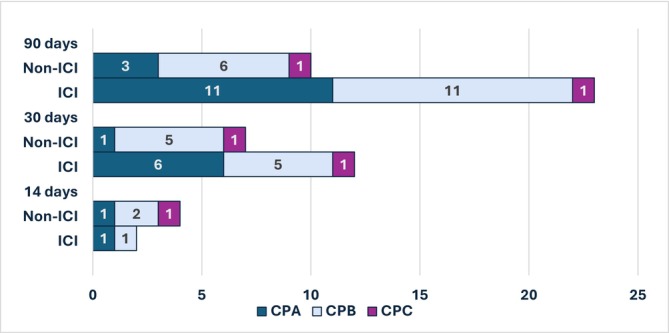
Last therapy stratified by Child‐Pugh Status. Figure 3 demonstrates the last therapy as ICI versus non‐ICI stratified by Child‐Pugh status at the time of last treatment, including 90, 30, and 14 days prior to death. The majority of patients received ICI compared to non‐ICI therapy within 90 or 30 days of death, including 12 patients with non‐Child‐Pugh A status 90 days prior to death and six patients with non‐Child‐Pugh A status 30 days prior to death.

Immune checkpoint inhibitors were given as a first‐line treatment in 62.0% of patients and included atezo/bev (*n* = 24), durva/treme (*n* = 9), nivo (*n* = 4), durva (*n* = 4), atezo (*n* = 2), and pembrolizumab (*n* = 1). In the second‐line setting, eight patients received ICI, and just three as third‐line treatment. The most common reasons for discontinuation of first‐line therapy were progression of HCC (39.2%, 20/51), worsening liver dysfunction (15.7%, 8/51), or decline in performance status (5.9%, 3/51). Grade 3/4 irAEs were reported in eight patients (CPA: *n* = 6; non‐CPA: *n* = 1; unknown: *n* = 1) and included hepatitis, colitis, myositis, acute interstitial nephritis, and pneumonitis. Of the eight patients with grade 3/4 irAEs, one was hospitalized for irAE management (myositis and hepatitis) within 14 days of death. Thirty‐two patients (43.7%) experienced a healthcare utilization event (hospitalization, ICU admission, or ED visit) within 90, 30, or 14 days of death. One patient was admitted at an outside hospital, so the cause of hospitalization is unknown; another patient was admitted for documented irAE, and one patient was admitted for nonspecific colitis, for which the differential was infectious versus irAE. The remaining 29 patients were admitted for complications of one or more of the following: decompensated cirrhosis (*n* = 12), infection (*n* = 5), cardiovascular complications (*n* = 4), complication of malignancy such as cancer‐related pain or progression of disease causing symptoms (*n* = 5), surgery (*n* = 2), assault (*n* = 1), generalized weakness (*n* = 1), and mucosal tear with hematemesis (*n* = 1). The ECOG PS at the time of initiation of last treatment was known in 27 patients who had a healthcare utilization outcome within 90 days of death: four were ECOG PS 0, 15 were ECOG PS 1, six were ECOG PS 2, and two were ECOG PS 3. Patients who received ICI versus non‐ICI therapy within 90, 30, and 14 days of death, stratified by CP status, are demonstrated in Figure 3.

## 4 Discussion

Our study showed no difference in EOL outcomes between patients who received ICI compared to those who received a non‐ICI agent as last systemic therapy. While patients receiving therapy toward the EOL (within 90 days of death) were more likely to be receiving ICI as systemic therapy, this did not translate to a decrease in AD documentation, GOC conversations, or palliative care or hospice referrals. Though there was no statistical difference in hospice duration, we did find a numerical increase in duration of days on hospice in patients who received non‐ICI (24.5 days) compared to ICI (10 days) therapy as the last treatment. The numerical difference highlights the clinical relevance as non‐ICI patients may have experienced more meaningful time with loved ones at the EOL compared to ICI patients. Our results are notable for high rates of documented GOC conversations (79.2% ICI and 85.2% non‐ICI) [17], palliative care (50.7% ICI and 69.2% non‐ICI), and hospice referrals (59.2% ICI and 87% non‐ICI) in both groups. Notably, early incorporation of palliative care in patients with solid tumors (including breast, prostate, colon, lung, and pancreas) has been increasing since 2010 (0.98% of patients), according to a retrospective SEER database review, but still remains low at only around 10.6% in 2019 [18]. Another database study including 1536 Native American HCC patients found that only 5.9% of patients received palliative care, the majority of those with stage III and IV disease [19]. Early palliative care integration may be dependent on socioeconomic factors, type of institution, and access to palliative care specialists, which may act as a barrier to incorporating palliative care early [20]. Unlike other solid tumors, where symptoms arise from the malignancy or treatment, symptomatology of HCC patients typically correlates with the degree of liver decompensation and often precedes their malignancy. In this way, HCC patients may represent a unique subgroup of oncology patients who would benefit from early incorporation of palliative care to address evolving symptom needs and conduct iterative discussions pertaining to patients' goals, values, and treatment preferences.

However, with regards to our secondary outcomes of healthcare utilization, a greater proportion of patients who received ICI as last therapy presented to the ED, were hospitalized, and/or were admitted to the ICU near the EOL compared to those who received non‐ICI as their last therapy, consistent with what has previously been noted in other solid tumors since the approval and incorporation of ICI [8, 9, 10]. Both clinicians and patients may not recognize that ICI treatment could lead to higher rates of healthcare utilization at the EOL. One study found that up to 58% of patients with HCC not enrolled in hospice died in the hospital (28% of those in the ICU), with 50% of those patients receiving invasive procedures such as mechanical ventilation [21]. Procedures and healthcare utilization during terminal hospitalizations can increase costs at the EOL, and data suggest that involvement of palliative care can both decrease procedures and healthcare costs [16]. Though we did not evaluate the impact of healthcare utilization on cost in our study, this is clinically important to recognize and discuss with patients when initiating ICI, given its tolerability and safety profile, to ensure treatment is concordant with patients' wishes.

These real‐world findings are especially relevant for non‐CPA patients who may be receiving more ICI at the EOL while simultaneously continuing to have serious illness conversations with the support and involvement of palliative care. Though patients with non‐CPA status often have increased mortality due to underlying liver dysfunction and so may receive ICI therapy for a shorter duration of time, prior data from the CPB cohort of Checkmate‐040 showed similar safety profiles in CPA compared to CPB patients with grade 3/4 treatment‐related AEs in 23% and 24%, respectively [5]. Similarly, a retrospective cohort study evaluating the safety of atezo/bev in CPB patients showed a grade 3/4 irAE rate of 17.4%. Of note, while no additional safety risks were identified in CPB patients in this study, those with CPB8/9 hepatic function had significantly worse survival compared to CPB7 [14]. Though our data cannot comment on the tolerability of ICI in non‐CPA patients due to the cohort size, our concern remains that prognosis is worse due to decreasing liver function and not the safety of ICI. Supporting this, a meta‐analysis reported similar rates of grade 3/4 treatment‐related AEs in CPB (12%) compared to CPA (11%) patients, and though prognosis was significantly shorter in CPB patients receiving ICI, this was not significantly associated with treatment‐related AEs in this cohort [12]. If patients with CPB status wish to pursue treatment, ICI may be an option insofar as patients are informed about the potential risk for toxicity and higher healthcare utilization, balanced with the potential for treatment efficacy. With that said, healthcare utilization outcomes may be altered if more patients with CPB status are receiving ICI toward the end of life, since studies have shown that patients with complications related to liver decompensation have clinic visits or hospitalizations seven times more per year than patients without decompensated liver disease [22]. The 1‐month hospital re‐admission rate after a decompensating event has been reported as up to 37% and remains an independent predictor of mortality in patients with liver disease [23]. We found this to be true in our patient cohort as the majority of patients with a healthcare utilization event within 90 days of death were admitted for a decompensating event related to cirrhosis. Another study evaluating EOL healthcare utilization in patients with liver disease found a significantly higher rate of hospitalization in the last 90 days of life, higher odds of inpatient death, and healthcare costs compared to patients who died of other chronic illnesses [24]. Thus, EOL outcomes related to ICI may be affected by the natural course of decompensated liver disease if more patients are receiving therapy perceived as more tolerable than TKIs.

Of note, our study shows a discrepancy between our defined EOL outcomes and healthcare utilization in patients receiving ICI therapy at the EOL. Efforts to increase palliative care integration, GOC documentation, and advance care planning are measures that typically correlate with reduced healthcare utilization and costs. However, we found that despite similarities in these outcomes in patients who received ICI versus non‐ICI, those patients receiving ICI as their last therapy remained high healthcare utilizers at the EOL. Several risk model tools, such as the LANS [25] and CRAFITY [26] scores, have been studied to predict response to ICI in patients with HCC, but to our knowledge, there are no needs assessment or risk stratification tools to re‐evaluate ICI use in patients with decompensating events. In other solid tumor types treated with ICI, such as melanoma, a prognostic risk stratification tool was studied to evaluate ICI use at the EOL and found increased ICI use in high‐risk patients, which was not associated with increased hospice referrals when compared to low‐risk patients [10]. This emphasizes the need for dedicated patient counseling and further risk stratification tools specific to HCC to predict high healthcare utilizers who might receive ICI close to death to ensure that patients' EOL care preferences and wishes are honored.

There are several limitations to this retrospective study. It was not powered to detect clinically meaningful differences in EOL outcomes, posing a risk of type II error. We were unable to evaluate healthcare costs and QOL patient‐reported outcomes while receiving ICI near the EOL, as this would help elucidate whether patients are truly tolerating this therapy well compared to non‐ICI therapy and provide a more comprehensive assessment. Our retrospective study is further limited by a small sample size and missing data within our single‐center cohort, which limits external validity (especially outside of the United States) and requires validation for subsequent prospective analyses with a larger patient population. For patients referred for consultation at our tertiary cancer center, there were limited data regarding EOL outcomes and healthcare utilization if they received care or were hospitalized at another facility. It is possible that patients had AD documented and GOC conversations with a local oncologist that were not documented within our EMR. Similarly, patients with healthcare utilization outcomes outside of our institution are unknown, and so our data may underestimate both healthcare utilization outcomes and hospice referrals. Lastly, since GOC conversations are not consistently found in a specific location in our EMR, content variability and subsequent documentation of GOC discussions may have impacted our results.

Future studies could focus on coordinating with community practices to capture patients who are primarily receiving therapy with their local oncologist. Additionally, multi‐center and larger cohorts could be explored to better characterize the heterogeneity within patients with HCC, allowing for subgroup analyses and stratification of outcomes based on ECOG PS, Child Pugh status, and timing of last ICI dose. Despite these limitations, since the treatment paradigm has changed within the last several years to include ICI, there has been little data reported on EOL outcomes and healthcare utilization in patients with HCC. The size of our study also confirms the real‐world use of ICIs in non‐CPA patients who are often excluded from clinical trials.

## 5 Conclusion

In summary, more patients are receiving ICI therapy within 90 days of death compared to TKI therapy, resulting in an increase in healthcare utilization for patients treated with ICI at the end of life. This may be due to increased real‐world ICI use in non‐CPA patients, given the overall tolerability and reported safety profile of ICI in this patient population. Though rates of palliative care involvement, goals of care discussions, and hospice referrals were not impacted based on receipt of ICI in our study, continued conversations surrounding HCC‐related mortality and potential for greater healthcare utilization are needed. Larger studies with multi‐center cohorts to increase generalizability, explore subgroup analyses, and better characterize CPB patients could be used to risk‐stratify patients with HCC receiving ICI. This information could then be used to develop a needs assessment or risk stratification tool to trigger re‐evaluation for ongoing systemic therapy and identify high‐risk patients. Furthermore, future studies that include evaluation of healthcare‐related costs and patient QOL or caregiver‐reported assessments may provide a more comprehensive assessment of the impact of ICI use near the end of life. Providers should be prompted to have iterative serious illness conversations regarding ICI continuation to inform and honor patients' wishes throughout their disease trajectories.

## Author Contributions

**Margaret C. Wheless:** conceptualization (equal), data curation (equal), investigation (equal), methodology (equal), writing – original draft (lead), writing – review and editing (supporting). **Anna‐Carson R. Uhelski:** conceptualization (equal), data curation (equal), investigation (equal), methodology (equal), writing – original draft (supporting), writing – review and editing (supporting). **Sarah K. Cimino:** data curation (equal), writing – review and editing (supporting). **Henry J. Domenico:** formal analysis (lead), writing – review and editing (supporting). **Sara F. Martin:** writing – review and editing (supporting). **Mohana B. Karlekar:** writing – review and editing (supporting). **Thatcher R. Heumann:** writing – review and editing (supporting). **Kristen K. Ciombor:** writing – review and editing (supporting). **Laura W. Goff:** writing – review and editing (supporting). **Rajiv Agarwal:** conceptualization (equal), investigation (equal), methodology (equal), supervision (lead), writing – original draft (supporting), writing – review and editing (lead).

## Ethics Statement

The study received ethical approval by the IRB at Vanderbilt University Medical Center (IRB# 231664), conducted in compliance with the U.S. Federal Policy for Protection of Human Subjects.

## Consent

Waiver of informed consent was approved to conduct this retrospective study. It was determined that the study poses minimal risk to participants. This study met 45 CFR 46.104 (d) category (4) iii for Exempt Review.

## Conflicts of Interest

KKC—Consultant/advisory board: Pfizer, Incyte, Exelixis, Bayer, ALX, Tempus, Taiho, Agenus, Merck, Beigene, Summit Therapeutics; Research grants: BMS, Array, Incyte, Nucana, Merck, Pfizer, Calithera, Genentech, Seagen, Syndax, Biomea. LWG—Consultant/advisory board: QED Therapeutics, Exelixis, Boehringer Ingelheim, Athenum Consulting, Relay Therapeutics, Merck, Bristol Myers Squibb Foundation, Jazz Pharmaceuticals, AstraZeneca; Research grants: BMS, Agios, ASLAN Pharmaceuticals, BeiGene, Bailea, Merck.

## Supporting information


**Table S1** Healthcare utilization outcomes within 90, 30, and 14 days of death stratified by receipt of immunotherapy.

## Data Availability

The data that support the findings of this study are available from the corresponding author upon reasonable request.
