# Supplementary material for: End‐Of‐Life Outcomes and Healthcare Utilization for Patients With Hepatocellular Carcinoma Who Received Immune Checkpoint Inhibition
Source: Cancer Med. 2025 Oct 10;14(19):e71293. doi: 10.1002/cam4.71293 (PMC12512345; doi:10.1002/cam4.71293)
Supplement: Supplementary file 1 — Table S1 Healthcare utilization outcomes within 90, 30, and 14 days of death stratified by receipt of immunotherapy. [file CAM4-14-e71293-s001.docx]

**Supplemental Table 1: Healthcare utilization outcomes within 90, 30, and 14 days of death stratified by receipt of immunotherapy**

| **Outcome**  *n* = 71 | **90 days** | **30 days** | **14 days** |
| --- | --- | --- | --- |
| **Therapy within**  Total:  Last therapy: ICI  Last therapy: non-ICI | 33/71 (46.5)  23/33 (69.7)  10/33 (30.3) | 18/71 (25.4)  12/18 (66.7)  6/18 (33.3) | 6/71 (8.5)  2/6 (33.3)  4/6 (66.7) |
| **ED visit**  Total:  Last therapy: ICI  Last therapy: non-ICI | 29/71 (40.8)  17/29 (58.6)  12/29 (41.4) | 27/71 (38.0)  15/27 (55.6)  12/27 (44.4) | 22/71 (30.1)  13/22 (59.1)  9/22 (40.9) |
| **Hospitalization**  Total:  Last therapy: ICI  Last therapy: non-ICI | 32/71 (45.1)  19/32 (59.4)  13/32 (40.6) | 30/71 (42.3)  17/30 (56.6)  13/30 (43.4) | 29/71 (40.8)  17/29 (58.6)  12/29 (41.4) |
| **ICU admission**  Total:  Last therapy: ICI  Last therapy: non-ICI | 6/71 (8.5)  4/6 (66.7)  2/6 (33.3) | 6/71 (8.5)  4/6 (66.7)  2/6 (33.3) | 6/71 (8.5)  4/6 (66.7)  2/6 (33.3) |

Supplemental Table 1: Healthcare utilization outcomes divided by 90, 30, and 14 days of death are stratified by their last treatment being ICI or non-ICI therapy. P-value for difference in proportion of patients with last therapy ICI between utilization outcome group at 90 days = 0.780, 30 days = 0.88, and 14 days = 0.64. P-values derived using Pearson’s chi-square test for equality of proportions. Abbreviations: ED, emergency department; ICI, immune checkpoint inhibitor; ICU, intensive care unit.
